# Faith Healing

**Published:** 1889-03

**Authors:** Parsons Shaw

**Affiliations:** Manchester, England.


ARTICLE III.

### FAITH HEALING.

BY PARSONS SHAW, D. D. S., MANCHESTER, ENGLAND.

As the effect of prayer upon pain has been definitely alluded to in the *Archives*, although the subject presents many difficulties, perhaps I shall not be out of place in offering some suggestions, and in putting forth what seems to me to be the line of thought which leads to the scientific investigation of this and kindred matters. I know there are a great many people so constituted that they instinctively resent almost any enquiry into what appears to them to be a mystery; but, on the other hand, there are so many more who reverently think that all things are governed by immutable laws which can not change or be changed, because they have their origin in a source that is divinely good and divinely true, that their views have a claim for hearing. Not that I am going to oppose the reality of what are known as "faith cures," but only to suggest how it may be possible for them to be effected in a perfectly natural manner. (N. B.) We are told on the best authority that we are to "Judge not according to appearance, but to judge righteous judgment." This is a fundamental law in all investigations, although it attracts but little attention. The ancient world thought the sun went round the earth, but we have since then judged *right*-eously,— that is, according to what is *right*,—and now know that we were deceived by appearances. And so in regard to a thousand other matters. We were all in a fog in regard to the origin of different species, and kindred subjects, until Darwin pointed out the way by which we could judge according to what was right. Is there nothing that will guide us in forming a right judgement in the matter of "faith healing?" I think there is, and that the subject is mastered when we thoroughly comprehend the scientific meaning of the two terms "objective" and "subjective." According to Worcester, *objective* is something "relating to the object of the thought, and not to the thinker." While "*subjective*" is something "relating not to the object, but the subject; relating to the conscious subject; not objective." And then, quoting Brande, a fuller explanation is given. "*Subjective* and *objective* are terms expressing the distinction which, in analyzing every intellectual act, we necessarily make between ourselves, the conscious *subject*, and that of which we are conscious, the *object*. For the distinction between subject and object, all-important in intellectual philosophy, and the neglect of which has been the cause of infinite confusion and perplexity, we are indebted to the schoolmen, from whom it was derived, through Wolfe and Leiblitz, by Kant and the modern German philosophers." The statement of this distinction by Kant seems to be that which is best known. But a host of thinkers did their best long before the German philosophers and Kant, to "make all things new" in the world of thought by showing that man saw everything from a wrong point when he took the objective view solely.

But intellectual growth is a very slow process, and it is only within the last few years that there has even begun to be a general acceptance among philosophers of the doctrine that the subjective view of natural phenomena is the one to reveal the secrets of their origin. The consequence is that the usual set and accepted phrases by which ideas are conveyed have not yet been framed for this doctrine, and it is, therefore, extremely difficult to convey an idea of subjective action. But I think that most dentists have the experience which will allow me to treat the matter with some degree of success. When a man (masculine) comes for consultation and is told he must have a tooth out, my experience is that he will either decline altogether or say "very well," or something like it, take hold of the chair, brace himself up, and submit to the operation at once. But what would be the effect if the dentist said nothing, took hold of the tooth without any warning to the patient and before he had "braced himself up?" He certainly would feel more pain. Then what takes place in the patient to make him bear the pain? It must be that some subjective influence has been at work upon his sensor nerves; for everything that is objective, the dentist and the forceps, remain the same. But when a woman comes under the same circumstances my experience is quite different. She will never willingly have the operation performed there and then, as the man does (if at all), but says she will wait until she can "make up her mind about it." Now, what does she mean by "making up her mind?" The answer is, as before, that she unconsciously brings to bear some subjective influence until she finds she can endure the pain; so that when she comes to have the operation performed she does not feel it as much as she otherwise would have done. Or, to put it in other words, in two different ways, according to the sex, by an effort of the will the sensor nerves seem to be acted upon and rendered less accute. On the other hand, we know that these nerves may be rendered abnormally accute by fright or any other exercises of the fear that great pain in is going to be given. The first intimation that I had that the will could produce absence of pain, was in what is now Portage City, in Wisconsin. I was there in 1852, and the Indians had just been removed. A party of both Winnebagos and Minominees had returned to the old place for a "drunk." The men got drunk on one day and the women on the other. Unfortunately it was "drunk day" for the women of both tribes, on the same day, and two squaws got into a fight and one was very badly wounded with a knife. Her wounds had been dressed as best her friends could manage, but she suffered great pain. She appeared not more than sixteen years old, and as she sat on a log giving way to moaning, some old Indian would go by and, giving a grunt, say something I could not and yet could understand, for her face would at once assume a calm expression as if she felt nothing, and this she would keep up for from ten to twenty minutes, when nature would again assert itself. I watched the girl for some hours, and learned a lesson never to be forgotten. *She had the power of controlling for a time the action of her sensor nerves.* And does not this explain the reported stoicism, or indifference to pain, the Indian is credited with? And why should not every living being be able to wholly control the action of his sensor nerves as he is undoubtedly able to partially control them? And in what but practice is the difference between controlling the action of tne sensor and the action of the motor nerves by an effort of the will? In after years I owned a wild mare caught on the plains, and this beast was another wonderful studv. She would go splendidly when it was the way she wanted to go, but would creep along in any other direction at a pace I do not care to risk my reputation for truth and veracity by attempting to describe. On one occasion I completely lost my temper and punished her severely; when, catching her eye, I saw the same expression I had noticed in the Indian girl. Anger turned to wonder, and I made that mare a study. From that, and much other experience, I became satisfied that animals can protect themselves from pain by subjective influences. Perhaps it will not be admitted that Dr. Livingstone, when shaken by the lion and had all the fear taken out of him, was under subjective influences; but his, and other similar cases, * and the curious habit of all the feline tribe in

* Sir Lyon Playfair, in a letter to Junius Henri Brown, author of a paper in the New York *Forum*, for October, says: "I have known three friends who were partially devoured by wild beasts under apparently giving their victims a good shake when they capture them helps my argument. The experience gained in the two above-named cases has proved of great value to me in administering the anæsthetic gas; for I find that unless the patient has "made up" his or her mind (or, in other words, produced more or less of what I have termed *mental* anæsthesia by an effort of the will) the gas is not a complete success. I never, therefore, undertake to push this agent upon any patient who is unwilling, or even uncertain, but wait until he has induced the right state of mind for taking it. In many ways does the subject exercise control over the action of the sensor nerves. How often do we count ourselves to sleep, when some disturbing state of the nerves keeps us awake? This process is akin to the one which produces what Dr. Braid, with whose experiments I was somewhat familiar, called *hypnotism*. Some produce this state more easily than others, and some are unable to produce it at all. If a susceptible subject places any object where he can concentrate his gaze upon it for a length of time, he will eventually fall into a hypnotic state. I have seen it produced on a public platform by the subject looking steadily at a coin placed in the palm of the hand. Hypnotism is also unconsciously produced in various other

---

hopeless circumstances of escape. The first was Livingston, the great African traveler, who was knocked on his back by a lion, which began to munch his arm. He assured me that he felt no fear or pain, and that his only feeling was one of intense curiosity as to which part of his body the lion would take next. The next was Rustem Pasha, now Turkish Ambassador in London. A bear attacked him and tore off part of his hand and part of his arm and shoulder. He also assured me that he had neither a sense of pain nor fear, but that he felt excessively angry because the bear grunted with so much satisfaction in munching him. The third case is that of Sir Edward Bradford, an Indian officer, now occupying a high position in the India Office. He was seized in a solitary place by a tiger, which held him firmly behind his shoulders with one paw and then deliberately devoured the whole of his arm, beginning at the end and ending at the shoulder. He was positive that he had no sensation of fear, and thinks that he felt a little pain when the fangs went through his hand, but is certain that he felt none during the munching of his arm."—*Scientific American.*

ways. When it is avowedly produced it is usually regarded by the spectator as some trick; but when it comes to certain people in favorite ways, they are apt to imagine it is but little short of, if not actually, some special revelation. Now, in my opinion, both the miracle lovers and the scoffers are wrong. There is not a doubt that a great deal of what goes under the name of "spiritualism," "magnetism," "clairvoyance," "hypnotism" and kindred names is conscious trickery, and always has been from the days long before when the Roman Augers laughed in each other's faces when they went through the routine of their calling down to the modern showman; but, as I understand this matter, there has always been and yet is, just enough of the genuine, but hitherto unexplainable phenomena coming in to puzzle and mislead the deceived and to give some confidence to the often unconscious deceivers. Hypnotism (I use this word, coined by Dr. Braid, not to provoke controversy over what has been called "Braidism," but simply because I think it best expresses the phenomena we are discussing) is simply a curious subjective influence which has been common to mankind for all time, but has been known under other names, and has been attributed to wrong causes. It has been employed to deceive the subject himself, and also by him to deceive others ever since man was an intelligent being. It has also (although unconsciously) been employed for many good purposes, not the least of which is the one named on page 576 of *Archives* for 1888. To one who realizes the power of subjective influences this case is no mystery, but a simple record of a daily occurrence. Not only can the repetition of prayer by the hypnotically disposed remove the pain of tooth-drawing, but it can dispel tortures to which this is but as the prick of a pin, and in the same way. In a fit of sudden anger a man has slain his brother; or, under the delirium of passion, a woman has committed her greatest sin; and when the reaction comes the horror of the situation produces an agony which is seemingly more than can be endured. At last, borne down with crushing penitence (a relief in itself to the overwrought nerves), the penitent goes into the church and, kneeling down, fixes the eye upon an image of a saint or the virgin and repeats, over and over again, in the monotonous way people repeat anything with which they are familiar, his "Pater Noster" or her "Ave Maria" until the excited nerves are reduced to quiet. I am not discussing the spiritual effect undoubtedly produced by penitence and prayer; but I contend that the outward calm that comes in such a case is a perfectly natural result akin to hypnotism, produced by the fixed gaze and the consequent abstraction of the mind. In short, natural results are always produced by natural causes.

When we see how overpowering is the subjective influences upon the objective surroundings, we are prepared to understand how pain is reduced and cures are effected by what appears not to be ordinary agencies. To begin with, a great deal of the illness in the world is the result of subjective influences. Then why is it strange to find they are cured in the same way? Thousands of illustrations could be given to show what I mean, but one will suffice. A medical friend once related to me the case of a girl who came one day while he was attending to hospital practice with her leg drawn up so much that she used a crutch, and also complained of much pain. All attempts to straighten it proved unavailing until the doctor put her under chloroform, when the leg was as right as ever. But just as soon as the patient began to be conscious, the leg began again to be drawn up, and was stiff and painful. The doctor looked solemn, and told her it was a very bad and a very curious case, and that she must come again the next day. Nothing loth to be made a heroine, the girl came as directed. In the mean time, the doctor had provided himself with a box; and as soon as the patient was under the chloroform, he straightened out her leg and fastened it into the box, so it could not be removed. When the patient began to recover there was a dreadful scene, and she would have done herself much damage if she had not been held. She declared that the pain was dreadful, which was very likely true. In time she got calmer, and then asked the doctor what he had done; upon which he explained. When she further asked how long he intended keeping her in that condition, he replied that he should do so until she promised to stop shamming and would get up and go to work. She resolutely held out for three days, when she gave up the contest and promised, and was discharged cured. Now, this girl was suffering from the effects of purely subjective influences. The probabilities are that at first she began by pretending to have a pain in her leg, and finding she got much sympathy, and an excuse for idleness, she kept the thing going until she fancied she really did have something the matter with it; and then a condition was easily induced in which it was gradually drawn up, and really gave her great pain when any attempt was made to straighten it. Fortunately for her, she fell into the hands of a man who knew how to cure her by the application of a little common sense which broke the spell into which she had voluntarily cast herself. But, let me ask, what would have been the result if this woman had, by some strange accident, dropped into one of those meetings where it is pretended that miraculous faith cures were effected? We know that in all these cases there is an immense amount of emotional excitement, sometimes displayed and sometimes suppressed. Indeed, it is all emotion with no pretense at the exercise of the judgment; and the more of this emotional faith that can be got up, the more certain is the prospect of results. As this girl had made herself a cripple by the combined action of her fancies and her will, she was a fit subject to be made well again by the same agencies. All that would have been required of her, if she had gone to some place as I have indicated, would have been to work herself up to the proper pitch of excitement until her mind had become diverted from her crutch and her stiff leg; and then, before she was aware of what she was doing, she would have put her foot to the floor,—a cured woman.* At such a proof of their powers, the believers in the miraculous would have made the world ring with this indisputable evidence; yet there would not have been the least trace of the miraculous in it. But, on the other hand, it must not be supposed that we have settled the matter when we have determined so much; for, however it had been done, *there would have been the cure*, and that fact would have to be accounted for. Therefore, instead of fighting over the fact of the cure, as do most of those who deny the miraculous influence, the real question is,—how could the cure have been effected? The answer to all such questions will be found, so I think, in a study of the subjective influences. The clinical influence of this potent power is so constantly manifested that one is sometimes almost led to the belief that it is the chief agent in the curative process. More people employ a medical man because they "have faith in him," than because of his learning and skill. It is not very far from the truth that as a man thinketh so is he; and that the objective surroundings are very much the ultimation of the ideal conceptions. The Mississippi valley was, a hundred years ago, a wilderness. But what is it to-day, and what has wrought this

* DEAR DOCTOR:—I saw the following in the "*Anglo-American Times*," only two days after posting you the article on cures by faith and anæsthesia by prayer. You will see that it is exactly on all fours with my supposition in the case of the girl, provided she had gone into a place where they cured "by faith:" "A faith cure that was witnessed by upwards of 200 persons, is told of in a despatch from Leesburg, Florida. S. B. Thompson, of Lady Lake, was for seventeen years a helpless cripple from a spinal trouble which doctors pronounced incurable. About ten days ago he was directed in a dream to pray in church for his cure and to get the congregation to do likewise. While carrying out the advice he thrice heard a voice saying: 'Arise and walk.' The third time the voice was quite distinct and positive, and he obeyed at once. Rising to his feet, he cried out, 'It is done,' and walked firmly and steadily out of the church, down the street and into his own house, shouting and praising God. He is now perfectly cured and walks easily. All Lady Lake, Leesburg and the intermediate country are agog over this apparent miracle, which is well and strongly attested."—P. S.

wonderful change? Certainly nothing of an objective character, for all these remain very much as before. There are the same rivers, lakes, mountains and skies. New Mexico is asking to be admitted into the Union as a State, and is refused as not being yet fit for the position. Yet, New Mexico was settled by white men long before Virginia and Massachusetts. Why is it that such a country, with all its advantages is in its present condition after three centuries of settlement, while a bleak and barren New England is teeming with population and wealth? There must be some subjective difference between the Indian, the Spaniard and the English, which is not difficult to find. In art the subjective influences are always apparent, and a man's *style* is the revelation of himself. No Frenchman or German can faithfully paint the portrait of an Englishman, and much less can an Englishman paint anybody but one of his own country. I was in Paris a few weeks since, and passed the new statue of Shakespeare, recently erected. One could see for whom it was intended, but it was not the real Shakespeare. The French artist did not have it in him to produce the image of one so intensely English; but give him one of his own countrymen to portray, and he would make a likeness. Catlin's portraits of Indians are only ugly white men dressed in Indian costume; and Power's Greek slave is a free-born Yankee girl, in spite of his theme and his model. This paper might be extended indefinitely, but I hope I have said enough to show that certain phenomena which have so long misled and puzzled the world, can be explained in a manner to show that they are governed by the same individual laws that control everything else.—*Archives of Dentistry.*

